# Endoscopic Submucosal Dissection for Early Gastric Cancer Exceeding Expanded Criteria—Long-Term Outcomes from the German ESD Registry

**DOI:** 10.3390/jcm13185538

**Published:** 2024-09-19

**Authors:** Kathrin Riedl, Andreas Probst, Alanna Ebigbo, Ingo Steinbrück, Hans-Peter Allgaier, David Albers, Matthias Mende, Michael Anzinger, Joerg Schirra, Viktor Rempel, Albrecht Lorenz, Siegbert Faiss, Ingo Wallstabe, Ulrike Denzer, Andreas Wannhoff, Franz Ludwig Dumoulin, Anna Muzalyova, Helmut Messmann

**Affiliations:** 1 Department of Gastroenterology, University Hospital Augsburg, 86156 Augsburg, Germany; 2 Medical Department, Evangelisches Diakoniekrankenhaus, 79110 Freiburg, Germany; 3 Department of Gastroenterology, Asklepios Klinik Hamburg Altona, 22763 Hamburg, Germany; 4 Department of Gastroenterology, Elisabeth-Krankenhaus Essen, 45138 Essen, Germany; 5 Department of Gastroenterology, Sana Klinikum Lichtenberg, 10365 Berlin, Germany; 6 Department of Gastroenterology, Barmherzige Brüder Krankenhaus München, 80639 München, Germany; 7 Medical Department II, University Hospital, Ludwig Maximilians-University, 80336 München, Germany; 8 Department of Gastroenterology, St. Anna Hospital, 44649 Herne, Germany; 9 Department of Gastroenterology, Helios Klinikum Berlin-Buch, 13125 Berlin, Germany; 10 Department of Gastroenterology, Asklepios Klinik Barmbek, 22307 Hamburg, Germany; 11 Department of Gastroenterology, Klinikum St. Georg, 04129 Leipzig, Germany; 12 Department of Gastroenterology, University Hospital Marburg, 35043 Marburg, Germany; 13 Department of Gastroenterology, Klinikum Ludwigsburg, 71640 Ludwigsburg, Germany; 14 Department of Gastroenterology, Gemeinschaftskrankenhaus Bonn, 53113 Bonn, Germany; 15 Institute of Digital Medicine, University Hospital of Augsburg, 86156 Augsburg, Germany

**Keywords:** early gastric cancer, endoscopy, endoscopic submucosal dissection, endoscopic resection, guideline criteria, expanded criteria

## Abstract

**Background and aims:** Endoscopic submucosal dissection (ESD) has become a standard treatment for early gastric cancer (EGC), often fulfilling guideline criteria (GC) or expanded criteria (EC). When lesions exceed the EC, surgical resection is recommended. However, a subgroup of these patients are not treated surgically. The aim of this study was to investigate the long-term follow-up of patients after ESD for EGC outside the EC (out of indication; OI). **Methods:** Patients who were included in the prospective German ESD registry were analyzed when ESD was performed for EGC. Patients were stratified in three groups according to histopathological features (GC, EC and OI). The results were evaluated in terms of patient characteristics, procedure characteristics and follow-up data. **Results:** Over a 48-month period, 195 patients from 14 German centers were included. In total, 71 lesions (36.4%) met the guideline criteria, 70 lesions (35.9%) corresponded to the expanded criteria and 54 lesions (27.7%) turned out to be OI. The R0 resection rate was significantly higher for the GC and EC groups than for the OI group (94.4% vs. 84.3% vs. 55.6%, *p* < 0.001). Additional surgery was not performed in 72% (39/54) of patients in the OI group. During a mean follow-up of 37 months, overall survival showed no significant difference between the EC and OI groups when endoscopic follow-up was performed without additional surgery (*p* = 0.064). **Conclusions:** The results show that a good long-term survival can be achieved after ESD for patients with OI lesions without additional surgery. The treatment decision has to be made on an individual basis, taking the patient’s comorbidities and the risk of surgical resection into account.

## 1. Introduction

Endoscopic resection (ER) has become a standard technique in the treatment of early carcinomas in the GI tract. An important point to consider in the adoption of ER as a curative treatment is the expected risk of lymph node metastasis (LNM) in the targeted lesion. For early gastric cancer (EGC), the Japanese Gastric Cancer Association defined guideline criteria (GC) in the 1990s. These guideline criteria are based on technical limitations of the former standard resection procedure of EMR. Resection was judged adequate in EGCs less than 20 mm in diameter, with the absence of ulceration, good or moderate differentiation, and the absence of submucosal or lymphovascular invasion [1].

Based on the findings of Gotoda et al. in 2000, subgroups of EGCs with a negligible risk of LNM were identified that did not fit the GC, and expanded resection criteria were defined (EC) [2]. The EC allowed the resection of well- or moderately differentiated lesions larger than 20 mm, ulcerated lesions ≤30 mm and lesions with submucosal invasion (<500 µm) with a size ≤30 mm. Further data also showed a low risk of LNM for small intramucosal undifferentiated-type EGCs [3,4,5].

Over the last two decades, ESD has become the treatment of choice for EGCs fulfilling GC or EC [6,7]. En bloc and R0 rates exceeding 90% have been reached [8,9,10]. Large studies show that this technique is safe [11,12] and leads to high curative resection rates with excellent overall survival and recurrence-free survival [13,14,15].

Lesions exceeding the EC are classified as out of indication (OI). For these lesions, surgical treatment is recommended to treat potential LNM. However, when lesions are diagnosed as OI after ER, a subgroup of patients are not treated surgically for different reasons (e.g., comorbidities or patient refusal). According to previous studies, a significantly higher risk of LNM and a worse prognosis are expected for these patients [2,16].

The aim of this multicenter study was to investigate the long-term follow-up of EGC patients after ESD with special regard to OI lesions.

## 2. Materials and Methods

The German ESD registry is a prospective, uncontrolled, multicenter study. Patients who underwent ESD for gastrointestinal neoplasia (esophagus, stomach, duodenum, colo, rectum) from January 2017 to December 2020 were included from 29 participating centers in Germany. The data were collected anonymously using an electronic case report form and managed in a central database at the University Hospital of Augsburg. Participating centers agreed to report all ESDs performed during the study period. Patients and lesions characteristics, procedural characteristics, complications, histopathologic assessments and follow-up data were reported. Ethics approval was granted by the ethics committee of the Ludwig Maximilian University of Munich, Germany (study ID: DRKS00011781). In addition, all participating centers received approval from their local institutional review boards.

For this study, all ESD procedures that were performed for EGCs were included from the registry. The decision to perform ESD for an early gastric neoplasia was at the discretion of each individual center. It is common practice in Germany to perform ESD for lesions fulfilling the guideline or expanded resection criteria according to morphological features (e.g., size, ulceration) and biopsy results (e.g., differentiation grade). The fact that a lesion was “out of indication” became apparent only after complete histopathological processing of the specimen. Surgery was then recommended to every patient who was fit for surgery.

### 2.1. Inclusion Criteria

1. Gastric cancer in the resection specimen after ESD;
2. Written informed consent to the ESD procedure after detailed information about ESD and alternative treatment strategies;
3. Written informed consent to enrolment in the database of the German ESD registry.

After the histopathological assessment of the ESD specimen, patients were stratified into three groups: EGC fulfilling GC (“guideline criteria group”, GC), EGC fulfilling EC (“expanded criteria group”, EC) and EGCs that were revealed to be OI by histological high-risk features that exceeded EC (“out of indication group”, OI). Patients with synchronous lesions were stratified according to the most aggressive lesion (e.g., patients with synchronous GC and EC lesions were included in the EC group). A sample size calculation was not performed within the design of the registry.

### 2.2. Outcome Criteria

The primary outcome parameter was overall survival in patients who did not undergo additional surgery after ESD. Secondary outcome parameters were procedural characteristics (R0 resection rate, adverse events), additional treatment after ESD (endoscopic or surgical), local recurrence, metachronous neoplasia and metastases (LNM or distant metastases).

Endoscopic follow-up examinations were performed 3, 6 and 12 months after curative resections and annually thereafter according to the German guideline [6]. In the EC and OI groups, further diagnostic measures (e.g., CT scans, ultrasounds) were performed at the discretion of the individual center without a standard protocol.

### 2.3. Definitions

ESD was performed as a standard ESD procedure or as a hybrid ESD. En bloc resection was defined as a resection of the target lesion in one piece. R0 situation was confirmed when histopathological assessment showed horizontal (HM) and vertical (VM) margins free of neoplasia. Curative resection was defined as R0 resection in lesions fulfilling the GC or EC by histopathological diagnosis. The GC include the following four characteristics: lesions size ≤2 cm, no ulceration, good or moderate differentiation, and absence of submucosal invasion. A lesion was assigned to the EC group when one of the four guideline criteria was not fulfilled. All lesions showing characteristics exceeding one or more of the expanded criteria were classified as “out of indication” [1]. Local recurrence was diagnosed when neoplasia was confirmed histopathologically at the resection site after an initial R0 situation. Bleeding was defined as a complication if a hemoglobin drop of more than 2 g/dl or clinical signs of bleeding were observed. Perforation was defined as a transmural injury of the gastric wall requiring endoscopic or surgical treatment [17].

### 2.4. Statistical Analysis

Categorical variables are presented as absolute numbers and percentages. Continuous metrics are shown as medians and interquartile ranges (IQR). Categorical data were compared using the chi-quadrat test. For comparisons of more than two groups, Bonferroni correction was performed to take into account multiple testing. Comparisons of the continuous data were performed using Kruskal–Wallis one-way tests followed by pairwise comparisons with correction for multiple testing. To compare the overall survival distribution of the three groups, Kaplan–Meier curves were used and log-rank analysis was performed. The significance level was set at 0.05. All calculations were performed using SPSS (Statistical Package for Social Sciences) version 28.0.

## 3. Results

**Figure 1:**
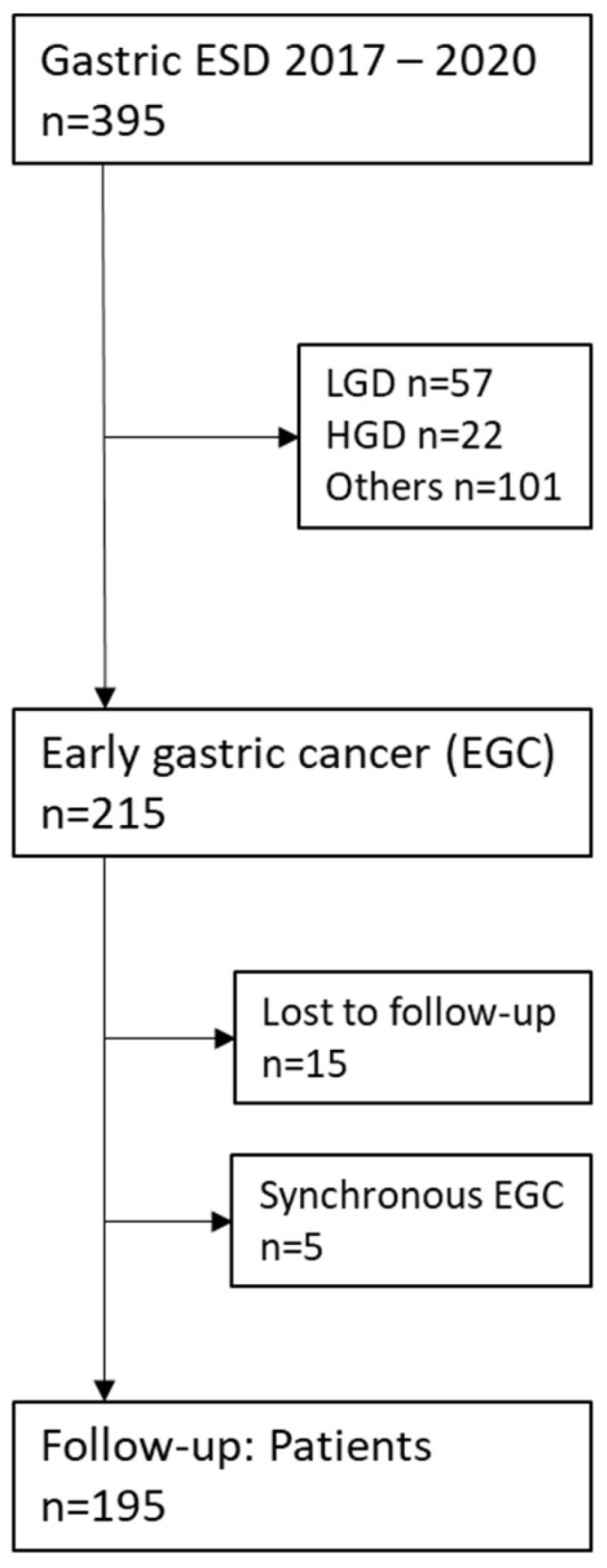
Patient inclusion. ESD: endoscopic submucosal dissection; EGC: early gastric cancer; LGD: low-grade dysplasia; HGD: high-grade dysplasia.

Based on the histopathological assessment, only patients with early gastric cancer were included. This resulted in a total number of 195 patients from 14 German centers (Figure 1).

### 3.1. Patient and Lesion Characteristics

We included 195 patients (127 men and 68 women) with a median age of 72.4 years (IQR [66–80]). Five patients showed synchronous EGC at the time of diagnosis (2 EGCs each) resulting in a total number of 200 EGCs. Patient and lesion characteristics are shown in Table 1.

**Figure 2:**
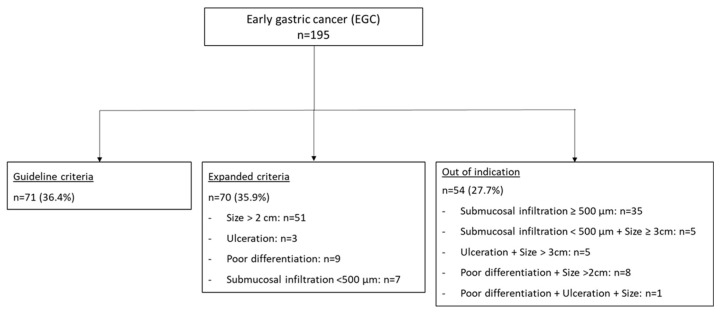
Included EGC: grouping according to histopathological assessment. EGC: early gastric cancer.

Notably, 35/54 lesions in the OI group were pT1b cancers with submucosal invasion exceeding 500 µm (64.8%; Figure 2). The other subgroups are described in Figure 1.

Prior to ESD, biopsies had been taken in 165/195 cases; LGD was found in 14 cases, HGD in 20 cases, and adenocarcinoma in 123 cases. In 8 patients, biopsies had not shown neoplastic tissue, and no biopsy had been taken in another 30 patients. In these patients, ESD was performed based on lesion morphology.

Data on underlying precancerous conditions such as autoimmune atrophic gastritis or HP gastritis were not recorded within the ESD registry.

### 3.2. Procedure Characteristics

The three groups were comparable regarding patient characteristics (age, sex, ASA classification; Table 2). En bloc resection was possible in 183 of 195 EGCs (93.8%), with no significant differences between the guideline criteria, the expanded criteria, or the out of indication lesions. The R0 resection rate was 80.0% for all ESDs. The R0 resection rate was significantly different for the three groups (94.4% for GC vs. 84.3% for EC vs. 55.6% for OI, *p* < 0.001). Complications included bleeding in 7.8% (15/195) and perforation in 4.7% (9/195) of patients. All complications were treated endoscopically. There was no significant difference between the three groups regarding the complication rate.

### 3.3. Follow-Up Data

#### 3.3.1. GC Group

**Figure 3:**
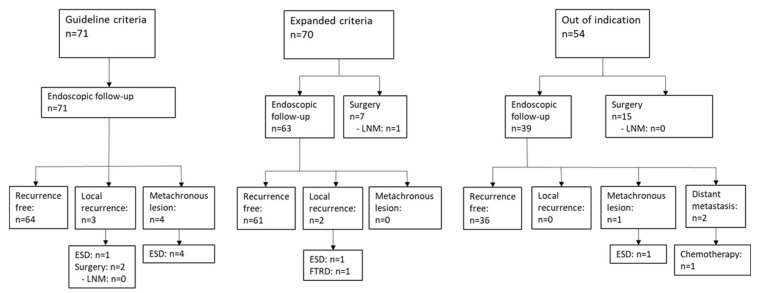
Follow-up of patients after endoscopic submucosal dissection of early gastric cancers. VM: vertical margin; HM: horizontal margin; ESD: endoscopic submucosal dissection; FTRD: full-thickness-resection; LNM: lymph node metastasis.

Notably, 64/71 patients were free of recurrence during follow-up (Figure 3 and Table 3). In three cases, a local recurrence was detected in the endoscopic follow-up (14, 19 and 57 months after the initial ESD). In one case, histopathological assessment of the initial lesion showed R0 resection; in two cases, there was an R1 situation on the horizontal margin. One patient was treated endoscopically (ESD); two patients underwent surgery.

Metachronous lesions were found in four cases (30, 37, 40 and 55 months after initial ESD) and were all treated successfully with repeated ESD.

#### 3.3.2. EC Group

In total, 7/70 (10%) patients underwent surgery. The lesion characteristics of EGC treated surgically after ESD were poorly differentiated in two cases, and displayed R1 resection on the HM in three cases. There was poor differentiation plus R1 at the HM in one patient, and superficial submucosal invasion in another patient.

The individual indications for surgery in these cases remain unclear, and 6/7 surgical specimens (85.7%) ruled out LNM.

In one case, LNM (4/28) was diagnosed. ESD had been performed for a mucosal, non-ulcerated, moderately differentiated EGC 40 mm in diameter. The specimen showed R1 resection on the HM. Distant metastases were diagnosed during further follow-up and the patient died nine months after the diagnosis of EGC. Metastatic gastric cancer was the presumed cause of death.

The remaining 63 patients entered follow-up. Sixty-one of them showed a recurrence-free follow-up, while local recurrence was detected in two cases. One patient developed recurrence after 19 months; the lesion was treated endoscopically with endoscopic full-thickness resection. Another patient developed local recurrences 3 and 34 months after initial ESD. Both lesions were treated with ESD.

#### 3.3.3. OI Group

In 54 patients, EGC exceeded the expanded criteria and surgery was recommended. Surgery was performed in 15/54 patients (27.8%). The surgical specimen showed residual cancer in one case. None of the surgical specimens showed LNM.

In the remaining 39 patients, endoscopic follow-up was performed without additional surgery. In one case, there was a metachronous lesion ten months after the initial ESD, which was R0-resected by repeated ESD.

In total, 2/39 patients (5.1%) who were not treated surgically developed distant metastases.

In one patient, hepatic metastases were diagnosed twelve months after ESD; the patient is currently receiving palliative chemotherapy. The ESD specimen had shown a deep submucosal invasive EGC (700 µm) that was R0-resected.

Another patient developed distant metastases. ESD had been performed for an ulcerated EGC with a maximum diameter of 52 mm, a submucosal invasion depth of 1000 µm and a positive VM. The grading was G2 and there was no lymphatic or vascular invasion (L0 V0). This patient died 46 months after the diagnosis of EGC.

### 3.4. Survival and Causes of Death

A total of 29 patients died during follow-up. One patient from the OI group died from metastatic gastric cancer 46 months after ESD.

Another patient from the EC group died from metastatic gastric cancer. In this case, surgery had been performed after ESD and LNM had been detected (4/28).

A total of 16 patients died from other causes (cardiovascular causes *n* = 5; nongastric cancer *n* = 4; others *n* = 7). In eleven patients, the cause of death remained unclear. The risk of death from gastric cancer was 1.9% in the OI group and 1.4% in the EC group. There was no gastric-cancer-related death in the GC group.

**Figure 4:**
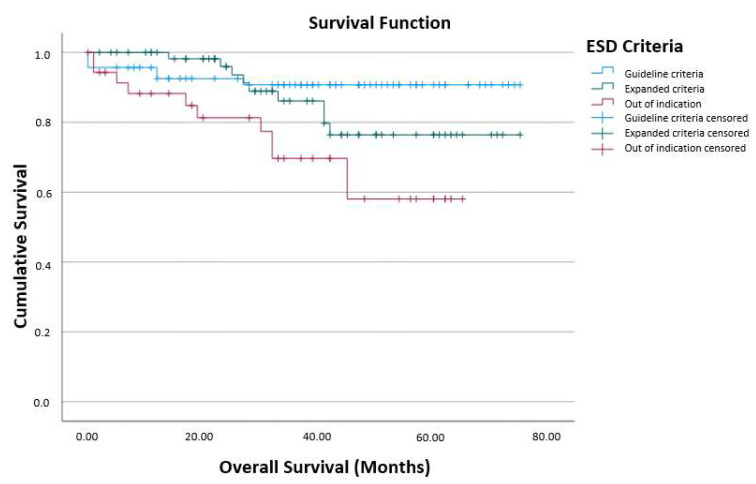
Overall survival for the different patient groups (*p* = 0.064 for the comparison of EC group vs. OI group).

For patients who underwent endoscopic follow-up without additional surgery, no significant difference in overall survival was seen between the EC group and the OI group (*p* = 0.064; Figure 4). Overall survival was comparable between the GC group and the EC group (*p* = 0.25). Overall survival was significantly different between the GC group and the OI group (*p* = 0.003). Median follow-up was 37 months for all patients (IQR [1,6,17–30,33–53]).

## 4. Discussion

ER is the standard treatment for EGC fulfilling GC or EC. ESD shows excellent R0 and en bloc resection rates and high rates of overall survival and recurrence-free survival [18,19]. However, data are scarce regarding the long-term follow-up of patients with lesions classified as OI after ESD. In our study, we investigated the long-term follow-up of these patients (OI group) in comparison to the GC and EC groups.

We included 195 patients with 200 EGCs resected with ESD in 14 German centers. Notably, 36.4% fulfilled the GC and 35.9% met the EC; 27.7% turned out to be OI after ESD. The three groups were of a similar size, whereas in other studies the proportion of EC lesions is higher [18].

Several studies have showed that en bloc resection rates and R0 resection rates exceed 90% for GC and EC [20,21]. Our study shows similar results, with an en bloc resection rate of 93.8% in total (GC group, 97.2%; EC group, 94.3%). The en bloc resection rate in the OI group was 88.9%, but the difference was not significant (*p* = 0.669).

In contrast, we observed significant differences between the groups regarding the R0 resection rate. In the GC group, an R0 resection rate of 94.4% was achieved, whereas the R0 resection rates in the EC group and the OI group were 84.3% and 55.6%, respectively. These results are comparable to other studies that also confirmed significant differences between R0 resection rates between GC and EC groups [22].

The main reason for R1 resection in the GC and EC groups was R1 resection at the horizontal margin (7.0% in the GC group, 17.1% in the EC group). In the OI group, a significantly higher R1 resection rate was observed at both the horizontal and vertical borders (HM 20.4%, VM 20.4%, *p* < 0.001). However, there was no significant difference in the occurrence of local recurrences (GC group 4.2%, EC group 2.9%, OI group 0.0%, *p* = 0.345). We also could not find a significant difference regarding metachronous lesions (GC group 5.6%, EC group 0.0%, OI group 1.9%, *p* = 0.130).

Overall, despite the significantly higher R1 resection rate, there was a high percentage of disease-free patients in the OI group (92.3%) that was not significantly worse than patients in the GC and EC groups (GC group 90.1%, EC group 96.8%, *p* = 0.304).

The complication rates were low and comparable for the three groups. The perforation rate was 4.7% (GC group 1.4%, EC group 7.1%, OI group 5.6%), whereas bleeding was reported in 7.8% of all cases (GC group 8.5%, EC group 8.6%, OI group 5.6%). Comparable studies show complication rates around 5% [23,24].

There was no statistically significant difference in gastric-cancer-related deaths between the three groups (GC group 0.0%, EC group 1.4%, OI group 1.9%).

In the OI group, only 15 of 54 patients (27.8%) underwent recommended surgery. There were no lymph node metastases in any surgical resection specimens. The occurrence of lymph node metastases in the OI group of our study is surprisingly low and lower than previous studies. This finding may be caused by the small sample size. The Japanese Gastric treatment guidelines [1] show 95% confidence intervals for the probability of LNM, depending on different combinations of histopathological criteria (size, ulceration, submucosal infiltration, differentiation). For example, for ulcerated lesions larger than 3 cm, the confidence interval reaches from 0.3% to 9.0%. For undifferentiated lesions >2 cm, the confidence interval is described as 1.0% to 6.0%. The 0% rate of LNM in all 15 patients of the OI group who underwent surgery does not contradict Gotoda’s findings [1].

Further statistical analysis showed no significant difference in overall survival between the GC and EC groups (*p* = 0.25) and between the EC and OI groups (*p* = 0.064). Significant overall survival difference was only found between the GC and OI groups (*p* = 0.003).

However, there are some limitations of our study that need to be taken into account. These include the small sample size and potential unmeasured confounding factors. There is no information about other comorbidities: only ASA status was recorded. There is variability in follow-up protocols across different institutions, including some short follow-ups, making the information regarding survival partly unreliable.

In our study, we were able to show that even in cases that exceed the histopathological features of the expanded criteria, a high overall survival rate can also be achieved even if no subsequent surgical resection is carried out. These results are interesting in an overall aging society, in which the proportion of patients is increasing for whom surgical therapy is not feasible due to comorbidities. Several studies showed a higher complication rate and a poorer quality of life after surgery was performed compared to ESD performed in cases of gastric cancer [13,23,25]. Shimada et al. showed that a severe comorbidity index, rather than gastric cancer, is the independent predictor of short-term survival after non-curative ESD without additional gastrectomy [26].

It is evident that older patients and patients with comorbidities are at risk of an increased perioperative complication rate and a loss of quality of life after surgery. Taking these risks into account, some Asian studies prefer endoscopic follow-up instead of surgery in cases of non-curative ESD [27,28,29,30].

When EGCs exceed the EC after ESD, further treatment decisions should be made on an individual basis, balancing the potential risk of LNM against the potential morbidity and mortality of invasive surgical procedures. If there are severe comorbidities and/or advanced age, endoscopic follow-up could be an alternative for these patients.

## Figures and Tables

**Table 1:** Patient and lesion baseline characteristics.

	Guideline Criteria	Expanded Criteria	Out of Indication	*p*-Value
Patient characteristics Age median ([IQR]), years Sex (male/female) ASA status (I/II/III/IV)	*n* = 71 70.2 (35–89) 45/26 18/38/15/0	*n* = 70 73.5 (46–87) 44/26 14/44/12/0	*n* = 54 74.1 (36–93) 38/16 8/28/16/2	0.045 0.551 0.135
Lesion characteristics Size ([IQR], (mm)) Maximum diameter Paris type Unknown 0-I 0-IIa 0-IIb 0-IIc 0-IIa + b 0-IIa + c 0-IIa + Is Ulcer Present/absent/unclear Lesion location Fundus Body Angle/antrum/pylorus	*n* = 71 11 (10–15) 0 (0.0%) 4 (5.6%) 33 (45.1%) 11 (15.5%) 5 (7.0%) 1 (1.4%) 15 (21.1%) 3 (4.2%) 0/71/0 13 (18.3%) 20 (28.2%) 38 (53.5%)	*n* = 70 27 (21–40) 0 (0.0%) 7 (10.0%) 27 (37.1%) 7 (10.0%) 9 (12.9%) 6 (8.6%) 11 (15.7%) 4 (5.7%) 3/67/0 11 (15.7%) 17 (24.3%) 42 (60.0%)	*n* = 54 30 (20–40) 2 (3.7%) 3 (5.6%) 14 (29.6%) 2 (3.7%) 7 (13.0%) 4 (7.4%) 16 (29.6%) 4 (7.4%) 10/42/2 15 (27.8%) 13 (24.1%) 26 (48.1%)	<0.001 0.068 <0.001 0.373
Histology Differentiation Good Moderate Poor Invasion depth Mucosal sm invasion < 500 µm sm invasion > 500 µm Lymphovascular invasion Lymphatic invasion Vascular invasion	49 (69.0%) 22 (31.0%) 0 (0.0%) 71 (100.0%) 0 (0.0%) 0 (0.0%) 0 (0.0%) 0 (0.0%)	38 (54.3%) 24 (34.3%) 8 (11.4%) 62 (88.6%) 8 (11.4%) 0 (0.0%) 0 (0.0%) 0 (0.0%)	11 (20.4%) 24 (44.4%) 19 (35.2%) 14 (25.9%) 5 (9.3%) 35 (64.8%) 9 (16.7%) 0 (0.0%)	<0.001 <0.001 0.002

**Table 2:** ESD procedure in 195 instances of early gastric cancer: resection rates and complications (HM: horizontal margin; VM: vertical margin).

	All Lesions (*n* = 195)	Guideline Criteria (*n* = 71)	Expanded Criteria (*n* = 70)	Out of Indication (*n* = 54)	*p*-Value
Procedure characteristics Resection rates En bloc resection R0 R1 HM R1 VM Piecemeal resection (Rx) Curative resection Median procedure time (IQR), minutes	183 (93.8%) 156 (80.0%) 27 (13.8%) 12 (6.2%) 12 (6.2%) 126 (64.6%) 80 (55–125)	69 (97.2%) 67 (94.4%) 5 (7.0%) 0 (0.0%) 3 (4.2%) 67 (94.4%) 66 (48.5–95)	66 (94.3%) 59 (84.3%) 12 (17.1%) 0 (0.0%) 5 (7.1%) 59 (84.3%) 83 (61–145)	48 (88.9%) 30 (55.6%) 11 (20.4%) 11 (20.4%) 4 (7.4%) 0 (0.0%) 97.5 (70.5–147.5)	0.669 <0.001 <0.001 <0.001 0.669 <0.001 <0.001
Complications Bleeding Perforation	15 (7.8%) 9 (4.7%)	6 (8.5%) 1 (1.4%)	6 (8.6%) 5 (7.1%)	3 (5.6%) 3 (5.6%)	0.507

**Table 3:** Follow-up of 195 patients with early gastric cancers treated by ESD.

	Guideline Criteria (*n* = 71)	Expanded Criteria (*n* = 70)	Out of Indication (*n* = 54)	*p*-Value
Surgery after ESD	0 (0.0%)	7 (10.0%)	15 (27.8%)	0.010
Endoscopic follow-up after ESD Local recurrence Metachronous lesion Distant metastasis Endoscopic retreatment Surgical retreatment	71 (100%) 3 (4.2%) 4 (5.6%) 0 (0.0%) 5 (7.0%) 2 (2.8%)	63 (90.0%) 2 (2.9%) 0 (0.0%) 0 (0.0%) 2 (2.9%) 0 (0.0%)	39 (72.2%) 0 (0.0%) 1 (1.9%) 2 (3.7%) 1 (1.9%) 0 (0.0%)	0.349 0.130 <0.05 0.469 0.178
Disease-free after endoscopic treatment	64/71 (90.1%)	61/63 (96.8%)	36/39 (92.3%)	0.304
Death From all causes From gastric cancer Other causes Unknown	7/71 (9.9%) 0/71 (0.0%) 4/71 (5.6%) 3/71 (4.2%)	10/70 (14.3%) 1/70 (1.4%) 5/70 (7.1%) 4/70 (5.7%)	12/54 (22.2%) 1/54 (1.9%) 7/54 (13.0%) 4/54 (7.4%)	0.116 0.532
Median follow-up (IQR), months	39 (23–54)	32.5 (19.25–50)	32.5 (7.5–55.5)	

## Data Availability

The original contributions presented in the study are included in the article, further inquiries can be directed to the corresponding author.

## Abbreviations

ASA	American Society of Anesthesiologists
EC	expanded criteria
EGC	early gastric cancer
ESD	endoscopic submucosal dissection
GC	guideline criteria
HGD	high-grade dysplasia
HM	horizontal margin
LGD	low-grade dysplasia
LNM	lymph node metastasis
OI	out of indication
Sm	submucosal invasion
VM	vertical margin
